# Anti-Inflammatory and Analgesic Effects of Pyeongwisan on LPS-Stimulated Murine Macrophages and Mouse Models of Acetic Acid-Induced Writhing Response and Xylene-Induced Ear Edema

**DOI:** 10.3390/ijms16011232

**Published:** 2015-01-06

**Authors:** You-Chang Oh, Yun Hee Jeong, Won-Kyung Cho, Jeong-Ho Ha, Min Jung Gu, Jin Yeul Ma

**Affiliations:** Korean Medicine (KM)-Based Herbal Drug Development Group, Korea Institute of Oriental Medicine, 461-24, Jeonmin-dong, Yuseong, Daejeon 305-811, Korea; E-Mails: ulivuli@kiom.re.kr (Y.-C.O.); runxi0333@kiom.re.kr (Y.H.J.); wkcho@kiom.re.kr (W.-K.C.); hahaha917@kiom.re.kr (J.-H.H.); guminjung@kiom.re.kr (M.J.G.)

**Keywords:** PW, HO-1, NF-κB, acetic acid-induced writhing response, xylene-induced mice ear edema

## Abstract

Pyeongwisan (PW) is an herbal medication used in traditional East Asian medicine to treat anorexia, abdominal distension, borborygmus and diarrhea caused by gastric catarrh, atony and dilatation. However, its effects on inflammation-related diseases are unknown. In this study, we investigated the biological effects of PW on lipopolysaccharide (LPS)-mediated inflammation in macrophages and on local inflammation *in vivo*. We investigated the biological effects of PW on the production of inflammatory mediators, pro-inflammatory cytokines and related products as well as the activation of nuclear factor kappa B (NF-κB) and mitogen-activated protein kinases (MAPKs) in LPS-stimulated macrophages. Additionally, we evaluated the analgesic effect on the acetic acid-induced writhing response and the inhibitory activity on xylene-induced ear edema in mice. PW showed anti-inflammatory effects by inhibiting the production of nitric oxide (NO), tumor necrosis factor-α (TNF-α) and interleukin-6 (IL-6) and interleukin-1β (IL-1β). In addition, PW strongly suppressed inducible nitric oxide synthase (iNOS), a NO synthesis enzyme, induced heme oxygenase-1 (HO-1) expression and inhibited NF-κB activation and MAPK phosphorylation. Also, PW suppressed TNF-α, IL-6 and IL-1β cytokine production in LPS-stimulated peritoneal macrophage cells. Furthermore, PW showed an analgesic effect on the writhing response and an inhibitory effect on mice ear edema. We demonstrated the anti-inflammatory effects and inhibitory mechanism in macrophages as well as inhibitory activity of PW *in vivo* for the first time. Our results suggest the potential value of PW as an inflammatory therapeutic agent developed from a natural substance.

## 1. Introduction

Inflammation is part of a complex biological immune response that occurs in vascular tissues due to harmful stimuli such as pathogens, cellular damage and irritants [1]. Inflammatory responses induce activation of immune cells such as macrophages, which play a critical role in the regulation of immune responses and secrete inflammatory mediators, including nitric oxide (NO), prostaglandin E_2_ (PGE_2_), tumor necrosis factor-α (TNF-α) and interleukin-6 (IL-6), after activation via lipopolysaccharide (LPS) stimulation [2,3,4,5]. NO and PGE_2_ are synthesized by inducible NO synthase (iNOS) and cyclooxygenase-2 (COX-2), respectively, and iNOS expression is closely related to the induction of heme oxygenase-1 (HO-1), a stress-inducible protein that catalyzes the oxidative degradation of heme. HO-1 expression is enhanced not only by free heme but also by various pro-inflammatory stimulants such as NO, LPS, cytokines, heavy metals and other oxidants [6,7]. Bilirubin and carbon monoxide, products of HO-1-induced heme degradation, inhibit NO secretion and reduce inflammation [8,9]. Thus, enhanced HO-1 production may result in reduced iNOS expression and a decreased inflammatory reaction [10].

TNF-α, IL-6 and IL-1β are important factors involved in various inflammatory processes that can be regulated by activation of nuclear factor kappa B (NF-κB). NF-κB is a transcription factor composed of p50 and p65 subunits that plays an important role in the expression of inflammatory genes and is involved in the pathogenesis of various inflammatory diseases [11]. Unstimulated NF-κB is present in the cytoplasm where it is attached to the suppressor protein inhibitor of NF-κB alpha (IκBα), and LPS activates NF-κB via degradation and phosphorylation of IκBα [12]. Activated NF-κB is translocated to the nucleus, where it binds to DNA promoters to induce production of inflammatory genes, including iNOS, COX-2, cytokines and chemokines [13,14]. The mitogen-activated protein kinases (MAPK) signaling pathway, involving extracellular signal-regulated kinase (ERK), p38 and c-Jun NH_2_-terminal kinase (JNK) MAPKs, plays an important role in relaying inflammatory information from the extracellular environment into the cytoplasm and nucleus [15]. MAPK is activated by phosphorylation to subsequently activate the NF-κB pathway and expression of *iNOS* genes.

Pyeongwisan (PW) is a traditional East Asian herbal medication currently prescribed for the treatment of symptoms associated with the digestive system. A recent study demonstrated that PW shows beneficial activity on perennial allergic rhinitis [16]. Another study showed that PW has a protective effect on gastric mucosal lesion membranes in mice [17]. In the present study, we evaluated the inhibitory effect of PW on inflammatory pathway activation induced by LPS and the influence on HO-1 induction in murine macrophages. Furthermore, we investigated *in vivo* the analgesic effects and anti-inflammatory activities of orally administered PW.

## 2. Results and Discussion

### 2.1. PW Is not Cytotoxic and Shows Inhibitory Effects on NO and Inflammatory Cytokine Production in RAW 264.7 Cells

**Figure 1:**
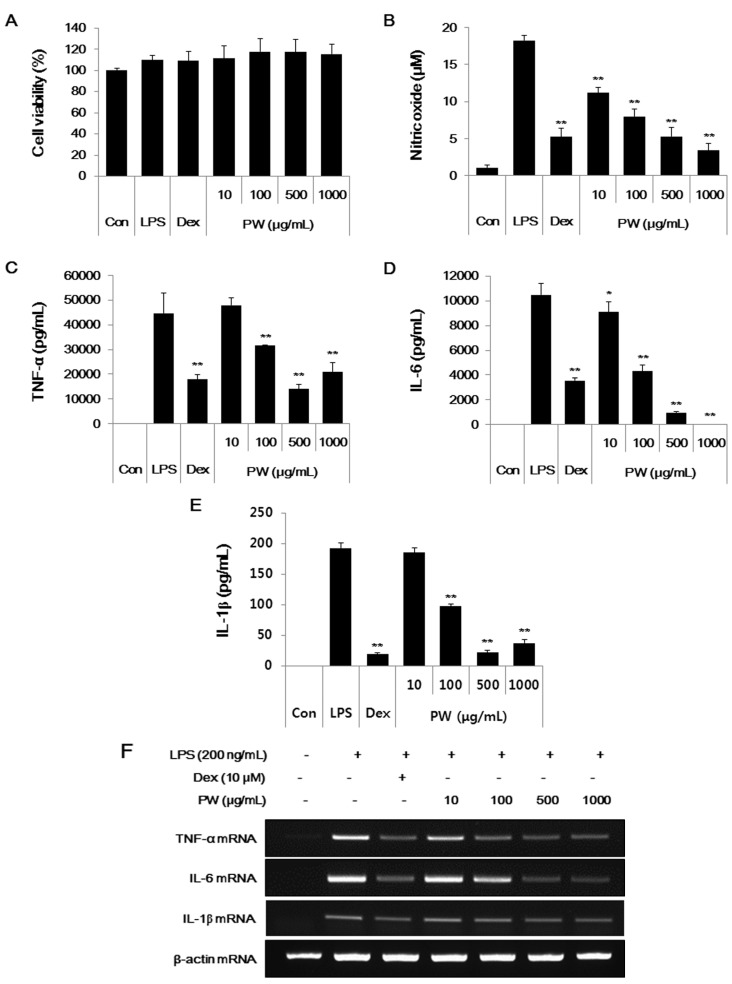

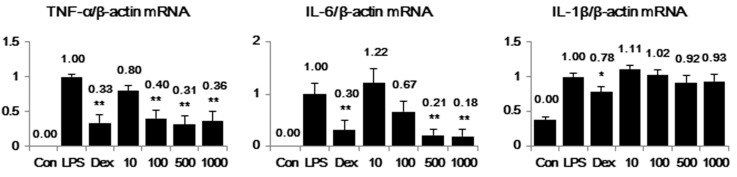
(**A**) Pyeongwisan (PW) cytotoxicity and (**B**–**F**) suppressive effect of PW on NO and cytokine production. RAW 264.7 cells were pretreated with PW for 30 min before incubation with LPS for (**A**–**E**) 24 h or (**F**) 6 h. (**A**) The cytotoxicity was determined using cell-counting kit (CCK); (**B**) The culture supernatant was analyzed for nitrite production; (**C**–**E**) Production of cytokines was measured using ELISA; and (**F**) mRNA levels were analyzed by RT-PCR. RNA values were quantitated using an i-MAX™ Gel Image Analysis System (Core Bio, Seoul, Korea). As a control, the cells were incubated with vehicle alone. Data represent means ± SE of duplicate determinations from three independent experiments. Con: control; Dex: Dexamethasone. *p < 0.01 and **p < 0.001 in comparisons of the LPS-stimulation value.

In this study, we evaluated the anti-inflammatory and analgesic activities of PW in macrophages and a mouse model. We first examined PW cytotoxicity at concentrations of 10–1000 μg/mL in macrophages. As shown in Figure 1A, PW was not cytotoxic even at 1000 μg/mL, indicating no toxicity in macrophages.

NO overproduction is associated with various inflammatory diseases [18,19], and NO inhibition can relieve inflammation; thus, we investigated the inhibitory effects of PW on NO production induced by LPS stimulation. Dexamethasone (10 μM), a well-known anti-inflammatory drug, was used as a positive control. As shown in Figure 1B, PW suppressed NO secretion in a concentration-dependent manner with statistical significance. In particular, PW (1000 μg/mL) inhibited NO secretion to a similar extent as observed with the positive control.

Furthermore, we examined the inhibitory effect of PW on the expression of other inflammatory mediators, TNF-α, IL-6 and IL-1β cytokines. Cytokine expression was analyzed using enzyme linked immunosorbent assay (ELISA) and reverse transcription PCR (RT-PCR) analysis. PW treatment strongly inhibited TNF-α cytokine and mRNA at concentrations of 500 μg/mL or more (Figure 1C,F). PW suppressed IL-6 cytokine and mRNA expression more than TNF-α in a concentration-dependent manner with statistical significance (Figure 1D,F). Additionally, PW strongly repressed both IL-1β cytokine production and mRNA expression in a dose-dependent manner, consistent with the other cytokine results (Figure 1E,F).

### 2.2. PW Strongly Inhibits LPS-Induced iNOS, but not COX-2, Expression and Induces HO-1 Induction

**Figure 2:**
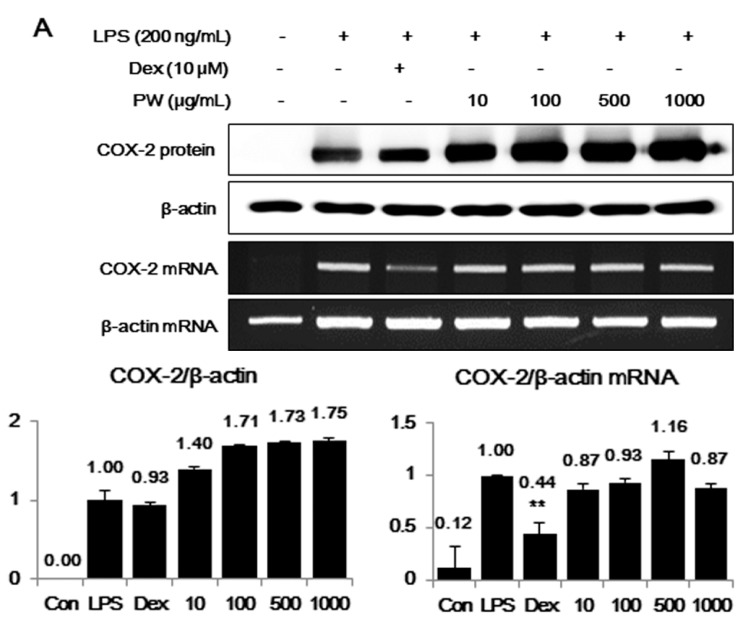

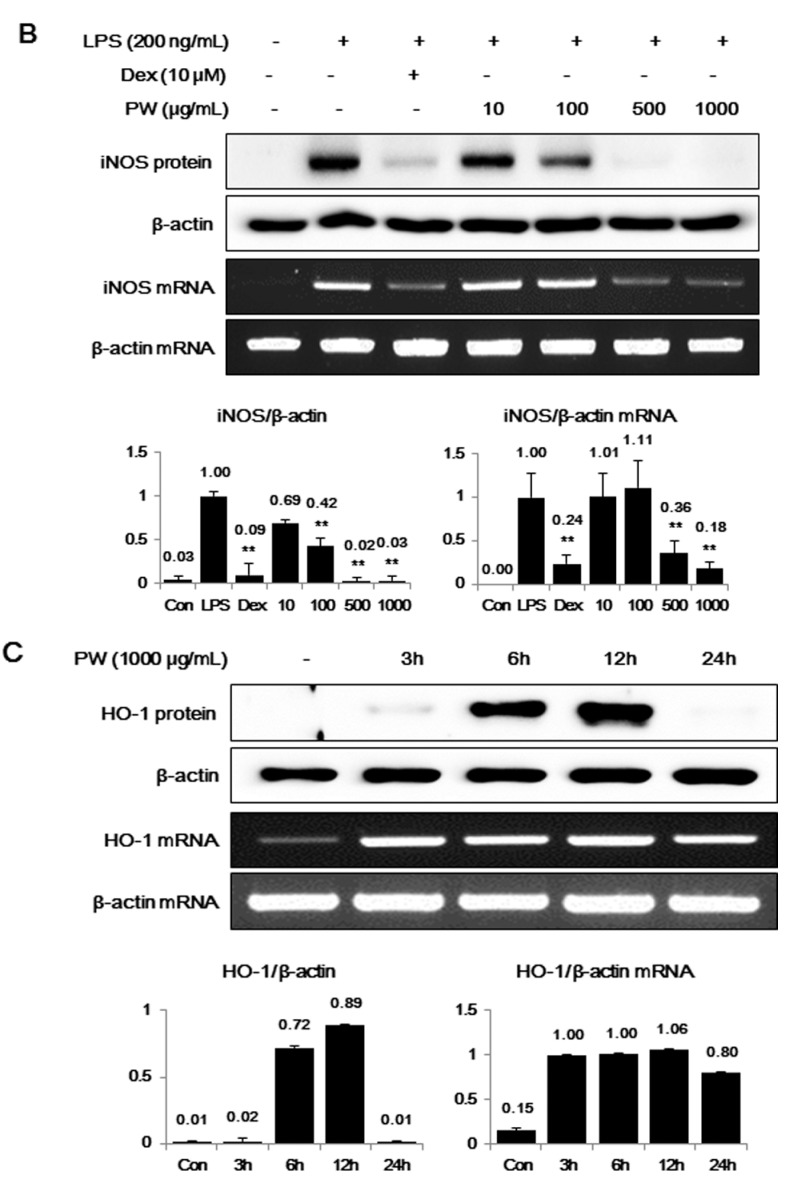

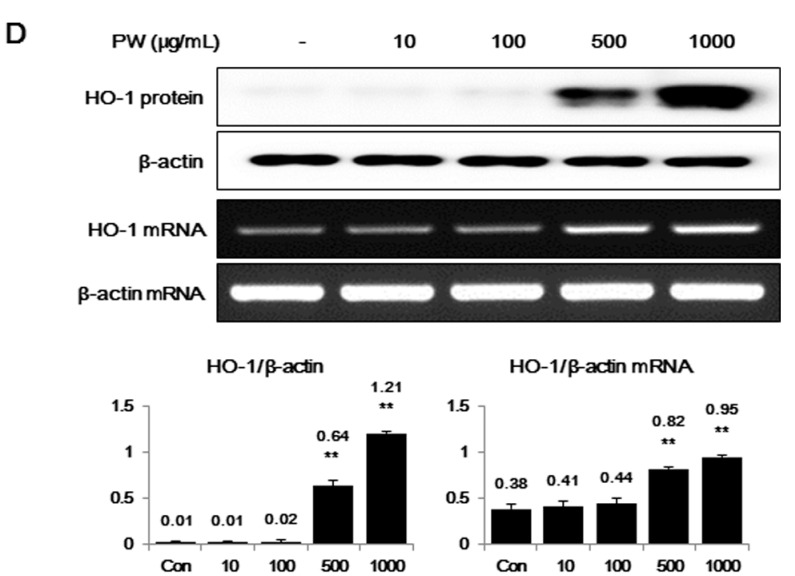
The effects of PW on (**A**) cyclooxygenase-2 (COX-2), (**B**) inducible NO synthase (iNOS) and (**C**,**D**) heme oxygenase-1 (HO-1) in macrophages. The cells were treated with (**A**,**B**) LPS alone or with LPS and PW for 24 h and (**C**,**D**) PW alone for the indicated periods. Protein levels were evaluated using Western blot analysis as described in the Materials and Methods and were quantitated using a Davinch-chemi™ Chemiluminescence Imaging System CAS-400SM (Core Bio, Seoul, Korea). The experiment was repeated three times independently and similar results were obtained. Con: control; Dex: Dexamethasone. ** *p* < 0.001 in comparisons of the (**A**,**B**) LPS-stimulation value or (**D**) non-treated control value.

COX-2 and iNOS expression were investigated next using Western blot analysis and RT-PCR. As presented in Figure 2A, PW did not show any suppressive effect on either COX-2 protein or mRNA expression. However, iNOS protein and mRNA expression was significantly inhibited by PW in a concentration-dependent manner (Figure 2B). The inhibition of PW on iNOS expression was closely related to the suppression of NO production.

The induction of HO-1 expression was due to a direct effect on iNOS expression [10]. Therefore, we investigated whether the inhibitory effect of PW on iNOS expression was associated with increased HO-1 production. Western blot and RT-PCR analyses revealed changes in HO-1 induction after PW treatment. First, we measured the expression of HO-1 at 3, 6, 12, and 24 h after 1000 μg/mL PW treatment. HO-1 protein and mRNA expression levels were highest at 12 and 3 h, respectively (Figure 2C). As shown in Figure 2D, PW induced HO-1 protein and mRNA expression at concentrations of 500 and 1000 μg/mL, respectively, in a concentration-dependent manner. These results indicated PW pretreatment induced HO-1 expression in RAW 264.7 macrophages and confirmed that it affected the inhibiting efficacy of NO and iNOS production.

### 2.3. PW Inhibits NF-κB Nuclear Translocation through Suppression of IκBα Degradation and Phosphorylation in LPS-Stimulated Macrophages

**Figure 3:**
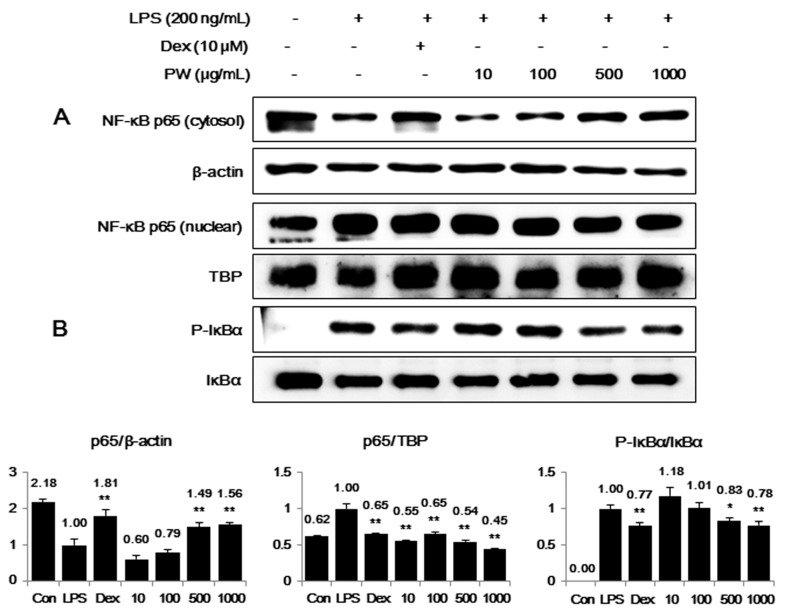
Inhibitory effect of PW on (**A**) NF-κB translocation into the nucleus and (**B**) IκBα phosphorylation. The cells were treated with LPS alone or with LPS and PW for 30 min (IκBα) or for 1 h (NF-κB). Protein expression in the cytosol or nucleus was evaluated using Western blot analysis. β-actin and TATA box-binding protein (TBP) were used for cytosolic and nuclear control protein, respectively. The experiment was repeated three times independently and similar results were obtained. Con: control; Dex: Dexamethasone. * *p* < 0.01 and ** *p* < 0.001 in comparisons of the LPS-stimulation value.

PW suppressed the inflammatory cytokines TNF-α, IL-6 and IL-1β in a concentration-dependent manner. NF-κB is a key transcriptional regulator associated with the cellular response to stimuli such as LPS [20,21,22] and plays an important role in cell viability and the expression of various inflammatory factors, including NO, inflammatory cytokines, PGE_2_ and iNOS [23,24,25]. To investigate whether the inhibitory effect of PW on the expression of cytokines and inflammatory factors was associated with NF-κB pathway activity, we examined the effects of PW on NF-κB activation by analyzing p65 translocation into the nucleus and the phosphorylation of IκBα in the cytosol. Western blot analysis showed 500 and 1000 μg/mL PW significantly repressed p65 translocation into the nucleus (Figure 3A). Additionally, as presented in Figure 3B, phosphorylation of IκBα was suppressed in a concentration-dependent manner; less IκBα was found consistently in the presence of the corresponding PW concentrations. These results suggest PW effectively inhibits LPS-induced NF-κB pathway activation by blocking nuclear translocation of NF-κB and IκBα phosphorylation. Additionally, these findings are consistent with previous studies showing the NF-κB response drives the expression of *iNOS*, *TNF-α* and *IL-6* genes [26,27,28].

### 2.4. PW Suppresses Activation of MAPKs via Phosphorylation after LPS Stimulation in Macrophages

**Figure 4:**
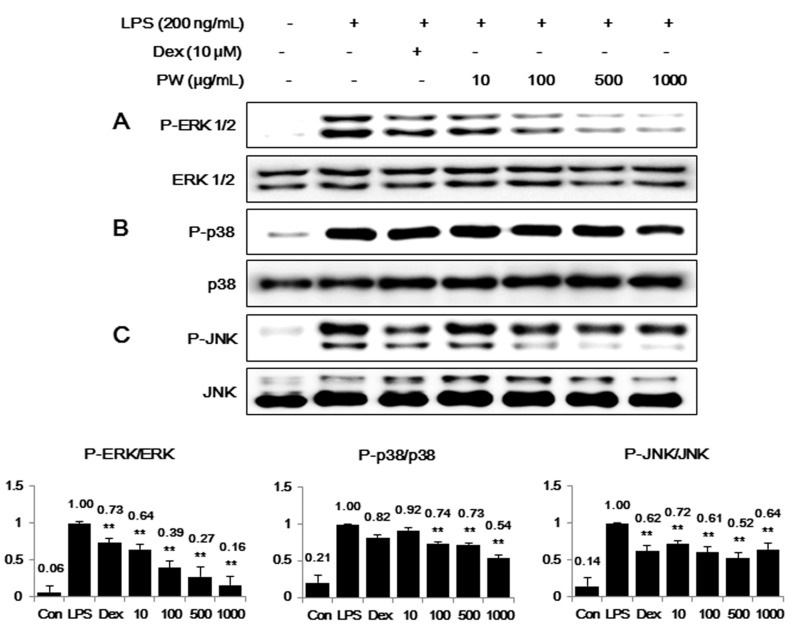
Effect of PW on MAPK phosphorylation in macrophages: (**A**) extracellular signal-regulated kinase (ERK); (**B**) p38 and (**C**) c-Jun NH_2_-terminal kinase (JNK). RAW 264.7 cells were treated with PW for 30 min before incubation with LPS for 30 min. Cell lysates were analyzed using Western blot analysis with specific antibodies. The experiment was repeated three times independently and similar results were obtained. Con: control; Dex: Dexamethasone. ** *p* < 0.001 in comparisons of the LPS-stimulation value.

Because phosphorylation-activated MAPKs play an important role in NF-κB pathway activation and are related to iNOS expression [29], we examined the inhibitory effect of PW treatment on activation of the MAPK pathway. The phosphorylation levels of MAPKs, including ERK1/2, p38 and JNK were evaluated. When RAW 264.7 cells were stimulated with LPS in the presence of PW, the level of phosphorylated ERK MAPK was significantly decreased in a dose-dependent manner (Figure 4A). Additionally, PW inhibited p38 or JNK phosphorylation at concentrations between 100 and 1000 μg/mL (Figure 4B,C), and the full forms of ERK, p38 and JNK were not affected by PW treatment. These results indicated the inhibitory effect of PW on the phosphorylation of MAPKs was directly related to inhibition of NF-κB activation and reduction of inflammatory factor production in RAW 264.7 cells.

### 2.5. PW Represses Inflammatory Cytokine Production in LPS-Stimulated Mouse Peritoneal Macrophage Cells

**Figure 5:**
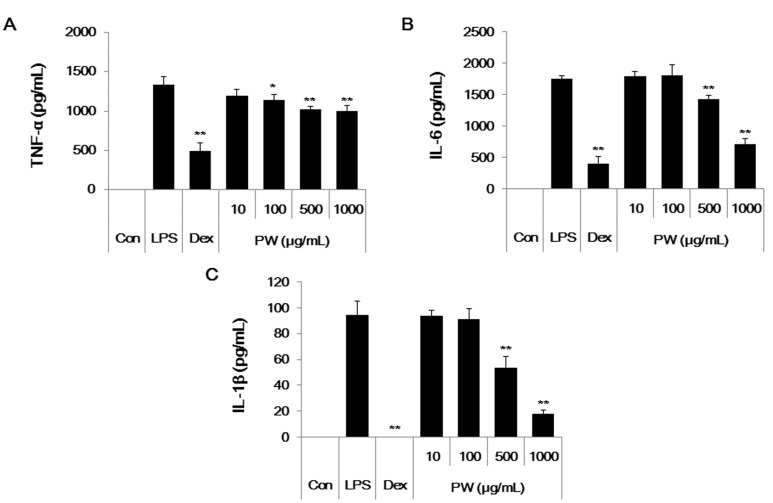
Effect of PW on (**A**) TNF-α, (**B**) IL-6 and (**C**) IL-1β cytokine production in mouse peritoneal macrophages. Cells were treated with PW for 30 min before incubation with LPS for 24 h. Production of cytokines was measured using ELISA. As a control, the cells were incubated with vehicle alone. Data represent means ± SE of duplicate determinations from three independent experiments. Con: control; Dex: Dexamethasone. * *p* < 0.01 and ** *p* < 0.001 in comparisons of the LPS-stimulation value.

Since we found strong anti-inflammatory effect of PW on LPS stimulated RAW 264.7 cells, we next tried to confirm its effect in mouse primary macrophage cells. We examined the levels of inflammatory cytokine secreted from the peritoneal macrophage cells treated with PW. As shown in Figure 5, consistent with the results obtained in RAW 264.7 cells, PW effectively suppressed inflammatory cytokine production including TNF-α, IL-6 and IL-1β by LPS stimulation in peritoneal macrophages without cytotoxicity (data not shown). These results indicate that PW strongly inhibits inflammatory response in the primary cells.

### 2.6. PW Showes Analgesic Activity on Acetic Acid-Induced Abdominal Writhing Response and an Inhibitory Effect on Xylene-Induced Mice Ear Edema

**Figure 6:**
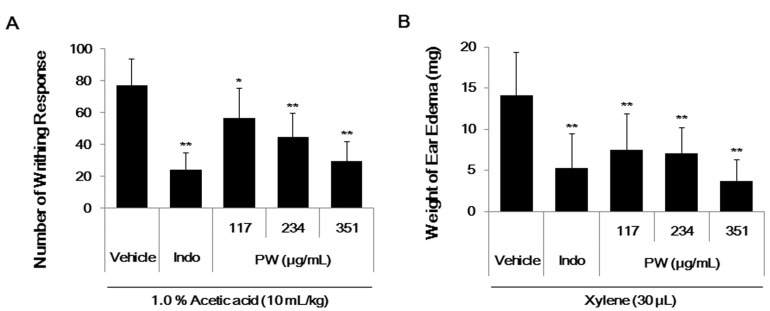
(**A**) The analgesic effect and (**B**) the anti-inflammatory effect of PW in male ICR mice. Each value represents the mean ± SE (*n* = 8). (**A**) The number of abdominal writhes was counted over 5–35 min after acetic acid intraperitoneal (i.p.) injection. * *p* < 0.01 and ** *p* < 0.001 as compared with the vehicle control + acetic acid group; (**B**) The weight of ear edema was measured 30 min after xylene application. ** *p* < 0.001 when compared with the vehicle control + xylene group. Indo; Indomethacin (10 mg/kg).

The anti-nociceptive activity of PW was assessed through experimental models of pain stimuli using the writhing test. The writhing response defined in abdominal writhing behavior was induced by acetic acid intraperitoneal (i.p.) injection, and indomethacin served as the analgesic positive control. An injection of acetic acid produced 77 ± 16.48 writhes in the vehicle control group. Treatment with indomethacin (10 mg/kg) resulted in a 69% inhibition in writhing when compared with the vehicle group. The oral administration of different doses of PW (117, 234 or 351 mg/kg) resulted in a significant reduction in the abdominal writhing responses after acetic acid injection (Figure 6A). A PW concentration of 351 mg/kg showed the maximal inhibition (62%), and the PW 50% effective dose (ED_5__0_) was 287.3 mg/kg.

As shown in Figure 6B, after xylene application, the weight of the mouse ear increased due to edema caused by an activated inflammatory reaction. However, oral administration of PW (117, 234 or 351 mg/kg) and indomethacin (10 mg/kg) significantly reduced the ear edema (63% inhibition). A PW concentration of 351 mg/kg showed the maximal inhibition (73%), and the PW ED_50_ was 251.4 mg/kg.

### 2.7. High Performance Liquid Chromatography (HPLC) Analysis and Previous Studies on the Main PW Constituents

**Figure 7:**
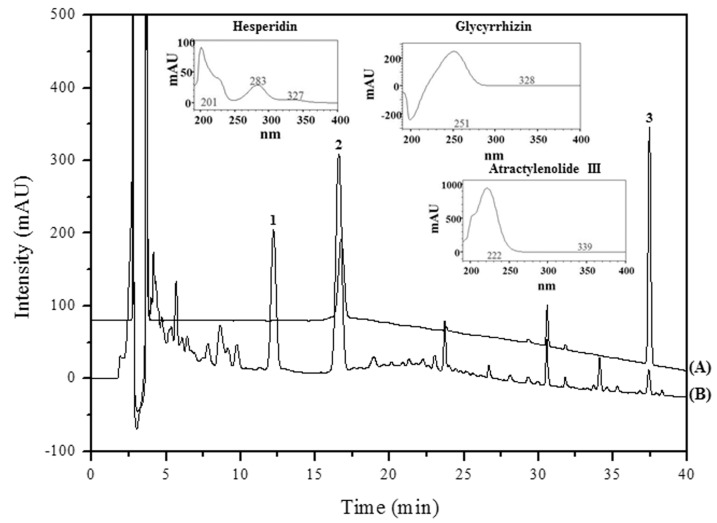
HPLC-DAD spectra and HPLC chromatograms of the main constituent compounds of PW. (A) Standard mixture and (B) PW at 230 nm. (1) hesperidin, 16.64 min; (2) glycyrrhizin, 29.74 min; (3) atractylenolide III, 37.46 min.

The results of HPLC-DAD analysis, using the mobile phases consisting of 0.1% aqueous trifluoroacetic acid (*v*/*v*) and pure acetonitrile with a gradient flow, showed satisfactory separation (Table 1). Optimal absorbance for all analytes (hesperidin, 201 nm; glycyrrhizin, 250 nm; atractylenolide III, 222 nm) was detected at 230 nm (Figure 7). Three of the retention time peaks (hesperidin, 16.64 min; glycyrrhizin, 29.74 min; atractylenolide III, 37.46 min) showed chromatograms of the reference components and a 60% methanol extract of PW (Figure 7). These compounds were identified qualitatively by comparing the retention times and UV wavelengths with those of standard compounds.

**Table 1:** HPLC conditions used for the analysis of PW.

Item	Condition		
Mobile Phase	Time (min)	Water (Containing 0.1% Trifluoroacetic acid (TFA))	Acetonitrile
	0	80	20
	10	80	20
	40	20	80
Flow Rate	1.0 mL/min		
Inject Volume	20 µL		
Column	OptimaPak C_18_ (4.6 × 250 mm, 5 µm, RS Tech Co., Daejeon, Korea)		
Column Temperature	35 °C		
UV Wavelength	230 nm		

A previous study reported that hesperidin downregulates NF-κB and its target molecules iNOS and COX-2 in mice [30]. Another study showed that glycyrrhizin inhibits NO and PGE_2_ production in a bimodal fashion [31]. Additionally, atractylenolide III was shown to inhibit LPS-induced TNF-α and NO production in macrophages [32]. These results suggest the anti-inflammatory activity of PW might be related to active components of PW, including hesperidin, glycyrrhizin and atractylenolide III.

## 3. Experimental Section

### 3.1. Materials and Reagents

The ingredients of the complete cell culture medium (Roswell Park Memorial Institute (RPMI) 1640 medium, fetal bovine serum (FBS) and antibiotics) were purchased from Lonza (Basel, Switzerland). The inflammatory inducer (LPS) and bovine serum albumin (BSA) were obtained from Sigma (St. Louis, MO, USA). A cell-counting kit (CCK) was purchased from Dojindo Molecular Technologies, Inc. (Kumamoto, Japan). Various primary and secondary antibodies for Western blot analysis were purchased from Cell Signaling Technology, Inc. (Boston, MA, USA). ELISA antibody sets for cytokine detection were obtained from eBioscience (San Diego, CA, USA). RNA extraction and DNA synthesizing kits were purchased from iNtRON (Sungnam, Korea) and Bioneer (Daejeon, Korea), respectively. Oligonucleotide primers were synthesized by Bioneer. Acetic acid and xylene were obtained from Merck (Darmstadt, Germany). The reference standard of glycyrrhizin was purchased from Tokyo Chemical Industry Co., Ltd. (Tokyo, Japan), atractylenolide III from Chem Faces (Wuhan, China) and hesperidin from the Ministry of Food and Drug Safety (Osong, Korea). The purity of all reference standards exceeded 98.0%. The high performance liquid chromatography (HPLC)-grade solutions, acetonitrile and trifluoroacetic acid were obtained from JT Baker Inc. (Philipsburg, NJ, USA). The triple distilled water (DW) was filtered through a 0.45-µm membrane filter (ADVANTEC, Tokyo, Japan) prior to analysis.

### 3.2. Preparation of the PW Herbal Decoction

PW is composed of six medicinal herbs (Table 2), all of which were purchased from Yeongcheon Herbal Market (Yeongcheon, Korea). All voucher specimens were deposited in an herbal tank, placed in 11,745 mL DW and extracted by heating at 115 °C for 3 h under high pressure (Gyeongseo Extractor Cosmos-600, Inchon, Korea). After extraction, the solution was filtered using standard testing sieves (150 μm) (Retsch, Haan, Germany), freeze-dried and kept in desiccators at 4 °C before use. The acquisition was 351 g, and the yield was 29.9%. The freeze-dried extract powder was then dissolved in DW and centrifuged at 14,000 rpm for 10 min, and the resulting supernatant was filtered (0.2-μm pore size) and kept at 4 °C prior to use.

**Table 2:** Herbal components and amount of PW decoction.

Herbs	Amount of Herbs (g)
Atractylodes Rhizome	400
Citrus Unshiu Peel	280
Magnolia Bark	200
Glycyrrhizae Radix et Rhizoma	120
Zingiberis Rhizoma Recens	74.5
Zizyphi Fructus	100

### 3.3. Cell Culture and Drug Treatment

RAW 264.7 cells were obtained from the American Type Culture Collection (ATCC, Manassas, VA, USA) and grown in complete RPMI 1640 medium. The cells were incubated in a humidified 5% CO_2_ atmosphere at 37 °C. To stimulate the cells, the medium was exchanged with fresh RPMI 1640 medium, and LPS (200 ng/mL) was added in the presence or absence of PW (10, 100, 500 or 1000 μg/mL) for the indicated periods.

### 3.4. Peritoneal Macrophage Isolation and Cell Culture

Male BALB/c mice were inoculated with 300 μL of sterile 3% sodium thioglycollate (Sigma). All mice were housed, 5 per cage at a room temperature and 12 h:12 h light/dark cycle. After three days the animals were killed and macrophages were harvested by washing their peritoneal cavity with 10 mL of ice-cold PBS. The cell suspension was centrifuged at 500× *g* for 5 min at 4 °C and the supernatant was discarded. The cell pellet was suspended in completed RPMI 1640 medium and incubated for 18 h to attach to the cell culture plate. To stimulate the cells, the medium was changed with fresh RPMI 1640 medium and LPS (1 μg/mL) [33] was added in the presence or absence of PW (10, 100, 500 or 1000 μg/mL) for the indicated periods.

### 3.5. Cell Viability Assay

Cytotoxicity was analyzed using a CCK. PW was added to the cells and incubated for 24 h at 37 °C and 5% CO_2_. CCK solutions were added to each well, and the cells were incubated for another 1 h. The optical density was then read at 450 nm using an ELISA reader (Infinite M200; Tecan, Männedorf, Switzerland).

### 3.6. Measurement of NO Production

NO production was analyzed by measuring nitrite in the supernatants of macrophages incubated with or without PW. The cells were pretreated with PW and stimulated with LPS for 24 h. Griess reagent (1% sulfanilamide, 0.1% naphthylethylenediamine dihydrochloride and 2.5% phosphoric acid) was added to the cultured supernatant and incubated at room temperature (RT) for 5 min [34]. The absorbance was read at 570 nm.

### 3.7. Determination of Cytokine Production

Secretion of the inflammatory cytokines TNF-α, IL-6 and IL-1β was analyzed using a mouse ELISA antibody set (eBioscience, San Diego, CA, USA). The inhibitory effect of PW was assessed using an ELISA reader at 450 nm absorbance.

### 3.8. Preparation of Cytosolic and Nuclear Extracts for NF-κB Detection

For isolation of cytosolic fractions, the cells were washed twice with cold PBS and incubated on ice for 10 min in lysis buffer (25 mM Tris-HCl (pH 7.4), 150 mM NaCl, 1 mM CaCl_2_, 1% Triton X-100, 1 mM PMSF and 10 μL/mL aprotinin), and the supernatant was collected after centrifugation at 15,000× *g* for 10 min at 4 °C. To prepare nuclear fractions, the cells were washed with 1 mL of ice-cold PBS, resuspended in 400 μL of ice-cold hypotonic low-salt buffer (10 mM HEPES-KOH (pH 7.9), 10 mM KCl, 2 mM MgCl_2_, 0.1 mM EDTA, 1 mM DTT and 0.5 mM PMSF), left on ice for 10 min, vortex-mixed and centrifuged at 15,000× *g* for 30 s. The pellets were resuspended in 50 μL of ice-cold high-salt buffer (50 mM HEPES-KOH (pH 7.9), 50 mM KCl, 1 mM DTT, 300 mM NaCl, 0.1 mM EDTA, 10% glycerol, and 0.5 mM PMSF), left on ice for 20 min, vortex-mixed and centrifuged at 15,000 g for 5 min at 4 °C to save nuclear fraction-containing supernatant. The fractions were stored at −80 °C before use.

### 3.9. Western Blot Analysis

The expression of various inflammatory proteins was evaluated using Western blot analysis according to standard procedures. The cells were pretreated with PW and stimulated with LPS for the indicated periods at 37 °C. After incubation, the cells were washed with ice-cold phosphate buffered saline (PBS), harvested and resuspended in radio immunoprecipitation assay (RIPA) lysis buffer (Millipore, Bedford, MA, USA) supplemented with a protease and phosphatase inhibitor cocktail (Roche, Basel, Switzerland). After the cell debris was discarded following centrifugation, the protein concentration was determined using Bradford reagent, and equal amounts of protein were subjected to sodium dodecyl sulfate-polyacrylamide gel electrophoresis (SDS-PAGE). After transferring the proteins onto a nitrocellulose membrane (millipore), the membrane was blocked with 3% BSA in Tris-buffered saline with 0.1% Tween 20 (TBS-T). The membrane was then incubated with each primary antibody at 4 °C overnight, followed by HRP-conjugated secondary antibodies. Information of various primary and secondary antibodies was listed in Table 3. Specific proteins were detected using enhanced chemiluminesence (Thermo Scientific, Hudson, NH, USA) or SuperSignal West Femto Chemiluminescent Substrate (Thermo Scientific).

### 3.10. RNA Extraction and Reverse Transcription-Polymerase Chain Reaction (RT-PCR)

Total RNA was isolated using an easy-BLUE™ RNA extraction kit (iNtRON, Sungnam, Korea) according to the manufacturer’s instructions. Total RNA was reverse transcribed into cDNA using AccuPower^®^ CycleScript RT PreMix (Bioneer, Daejeon, Korea). The specific primers used in polymerase chain reaction (PCR) are listed in Table 4. The following PCR conditions were applied for evaluating TNF-α, IL-6, IL-1β, COX-2, iNOS, HO-1, and β-actin expression: 35 cycles of denaturation at 94 °C for 30 s, annealing at the temperature indicated in Table 3 for 30 s and extension at 72 °C for 30 s [34,35,36,37,38].

**Table 3:** Primary and secondary antibodies use for Western blot analysis.

Antibody	Corporation and Product Number	Dilution Rate
COX-2	Cell signaling technology #4842 (Beverly, MA, USA)	1:5000
iNOS	Cell signaling technology #2977	1:1000
HO-1	Santa Cruz biotechnology #SC-10789 (Santa Cruz, CA, USA)	1:500
NF-κB p65	Cell signaling technology #3034	1:1000
P-IκBα	Cell signaling technology #2859	1:1000
IκBα	Cell signaling technology #4814	1:1000
β-actin	Santa Cruz biotechnology #SC-47778	1:5000
TBP	Santa Cruz biotechnology #SC-33736	1:1000
P-ERK	Cell signaling technology #4377	1:1000
ERK	Cell signaling technology #9102	1:1000
P-p38	Cell signaling technology #9211	1:1000
p38	Cell signaling technology #9212	1:1000
P-JNK	Cell signaling technology #9251	1:1000
JNK	Cell signaling technology #9252	1:1000
Secondary anti-mouse	Thermo scientific #31437 (Hudson, NH, USA)	1:5000
Secondary anti-rabbit	Thermo scientific #31463	1:5000

**Table 4:** Primers used for RT-PCR analysis.

Target Gene		Primer Sequence		Annealing Temperature	
*TNF-α*		F: 5'-AGCACAGAAAGCATGATCCG-3'		55 °C	
		R: 5'-GTTTGCTACGACGTGGGCTA-3'			
*IL-6*		F: 5'-CATGTTCTCTGGGAAATCGTGG-3'		58 °C	
		R: 5'-AACGCACTAGGTTTGCCGAGTA-3'			
*IL-1β*		F: 5'-TGCAGAGTTCCCCAACTGGTACATC-3'		64 °C	
		R: 5'-GTGCTGCCTAATGTCCCCTTGAATC-3'			
*COX-2*		F: 5'-CACTCAGTTTGTTGAGTCATTC-3'		45 °C	
		R: 5'-GATTAGTACTGTAGGGTTAATG-3'			
*iNOS*		F: 5'-AGCCCAACAATACAAATGACCCTA-3'		56 °C	
		R: 5'-TTCCTGTTGTTTCTATTTCCTTTGT-3'			
*HO-1*		F: 5'-TGAAGGAGGCCACCAAGGAGG-3'		62 °C	
		R: 5'-AGAGGTCACCCAGGTAGCGGG-3'			
*β-actin*		F: 5'-ATGAAGATCCTGACCGAGCGT-3'		58 °C	
		R: 5'-AACGCAGCTCAGTAACAGTCCG-3'			

F, forward; R, reverse.

### 3.11. Experimental Animals

Male ICR mice (30 ± 3 g) used for the experiments were obtained from Samtako BioKorea (Osan, Korea). All animals were maintained in a room with controlled temperature under a 12-h light/12-h dark cycle, with food (SCF Co., Ltd., Seoul, Korea) and water provided ad libitum. All mice were acclimatized for at least 7 days prior to beginning the experiments. All animal studies were performed according to the Guide for the Animal Care and Use Committee of the Korea Institute of Oriental Medicine (reference numbers: 14-037 and 14-049).

### 3.12. Acetic Acid-Induced Abdominal Writhing Response

The male ICR mice were divided into five groups of eight mice each and fasted overnight. Mice were treated per oral administration (p.o.) with vehicle 0.05% methyl cellulose (MC, 10 mL/kg), indomethacin (10 mg/kg) or PW (117, 234 or 351 mg/kg) 1 h before administration of a 1.0% acetic acid aqueous solution (10 mL/kg, i.p.) [39,40,41]. The animals were then placed in a transparent plexiglass chamber, and the number of writhes was counted under continuous observation for 30 min beginning 5 min after the acetic acid injection. A writhe was defined as an abdominal wall contraction and pelvic rotation followed by hind limb extension.

### 3.13. Xylene-Induced Mice Ear Edema

The male ICR mice were divided into five groups of eight mice each. Animals were treated (p.o.) with vehicle (0.05% MC, 10 mL/kg), indomethacin (10 mg/kg) or PW (117, 234 or 351 mg/kg). One hour after the treatment, ear edema was induced by applying 30 μL xylene on the inner surface of the right ear [42,43]; the left ear was used as control. After 30 min, the mice were sacrificed, and both ears were removed and weighed. Edema was defined as the difference in weight between the two ears.

### 3.14. Chromatographic Separation

All separations were performed using a Hitachi HPLC system (Hitachi, Tokyo, Japan), consisting of an L-2130 pump, L-2200 auto-sampler, L-2300 column oven and L-2455 diode array UV/VIS detector (DAD). The data processor used was EZchrom Elite software for Hitachi (Tokyo, Japan). The analytical column was OptimaPak C18 (4.6 × 250 mm, 5 µm, RS tech Co., Daejeon, Korea). The mobile phases were 0.1% aqueous trifluoroacetic acid (*v*/*v*, solvent A) and pure acetonitrile (solvent B). The gradient flow was 20% solvent B (0–10 min) and then 20%–80% (10–40 min). The column temperature was maintained at 35 °C. Analysis was performed at a flow rate of 1.0 mL/min with DAD detection at 230 nm and an injection volume of 10 µL.

### 3.15. Preparation of Standard Solutions and Samples

The standard stock solutions of atractylenolid III, hesperidin and glycyrrhizin were prepared by dissolving 0.2 mg of each standard in 1 mL 60% methanol to yield a final concentration of 200 µg/mL. To prepare the analytical samples, 10 mg PW extract in 1 mL DW was extracted by ultrasonication and filtered through a 0.2-μm syringe membrane filter from Whatman Ltd. (Maidstone, UK) before injection into the HPLC system for analysis. All standard stock and sample solutions were stored at −4 °C before analysis.

### 3.16. Statistical Analysis

The results were expressed as means ± standard error (SE) for all experiments. Statistical significance between the treated groups and the negative control was determined by Student’s *t*-tests. Each experiment was repeated at least three times to yield comparable results, and *p*-values <0.01 and <0.001 were considered to indicate statistical significance.

## 4. Conclusions

In conclusion, PW had a strong inhibitory effect on NO secretion, inflammatory cytokine production and iNOS expression in LPS-stimulated RAW 264.7 cells. These effects were due to inhibition of NF-κB activation through suppression of IκBα degradation and blockade of MAPK phosphorylation. Additionally, the influence of PW on HO-1 expression affected the suppression of inflammatory factors. Also, PW contains the inhibitory effect on the inflammatory cytokine production including TNF-α, IL-6 and IL-1β in mouse primary cells. Furthermore, PW showed an analgesic effect and inhibitory activity on acetic acid-induced abdominal writhing response and xylene-induced ear edema in an *in vivo* mice model. These results suggest that PW could be developed as a new anti-inflammatory agent derived from natural products.
